# Marijuana Use and Depressive Symptoms; Gender Differences in African American Adolescents

**DOI:** 10.3389/fpsyg.2018.02135

**Published:** 2018-11-16

**Authors:** Shervin Assari, Ritesh Mistry, Cleopatra Howard Caldwell, Marc A. Zimmerman

**Affiliations:** ^1^Department of Psychiatry, School of Medicine, University of Michigan, Ann Arbor, MI, United States; ^2^Center for Research on Ethnicity, Culture and Health, School of Public Health, University of Michigan, Ann Arbor, MI, United States; ^3^Department of Health Behavior and Health Education, School of Public Health, University of Michigan, Ann Arbor, MI, United States

**Keywords:** marijuana use, depression, African American, gender, adolescents

## Abstract

**Introduction:** This study aimed to examine gender differences in the bidirectional associations between marijuana use and depressive symptoms among African American adolescents. The study also tested gender differences in the effects of socioeconomic status, maternal support, and friends’ drug use on adolescents’ depressive symptoms and marijuana use.

**Methods:** This is a secondary analysis of the Flint Adolescent Study (FAS). Six hundred and eighty one African American adolescents (335 males and 346 females) were followed for 3 years, from 1995 (mean age 16) to 1997 (mean age 19). Depressive symptoms (Brief Symptom Inventory) and marijuana use were measured annually during the follow up. We used multi-group latent growth curve modeling to explore the reciprocal associations between depressive symptoms and marijuana use over time based on gender.

**Results:** Baseline marijuana use was predictive of an increase in depressive symptoms over time among male but not female African American adolescents. Baseline depressive symptoms were not predictive of an increase in marijuana use among male or female adolescents.

**Conclusion:** Study findings suggest that male African American adolescents who use marijuana are at an increased risk of subsequent depressive symptoms. Interventions that combine screening and treatment for marijuana use and depression may be indicated for African American male adolescents.

## Introduction

Overall, African American individuals have lower rates of major depressive disorder (MDD) (Blazer et al., 1994; Kessler et al., 1994; Williams et al., 2007) but higher levels of depressive symptoms (Blazer et al., 1998) relative to White Americans. Possibly due to a lower access to the health care system and a higher stigma associated with mental health problems (Menke and Flynn, 2009), depression tends to be more chronic, severe, and disabling for African American individuals compared to White Americans (Jones-Webb and Snowden, 1993; Williams et al., 2007).

Marijuana is one of the most frequently used drugs by African American adolescents (CDC, 1998; Miech et al., 2017). While African American adolescents are less likely to use marijuana compared to their White counterparts, the consequences of drug use may be more devastating for them (Wallace and Bachman, 1993). Given their lower access and adherence to treatment (Cook and Alegría, 2011; Mericle et al., 2012; Schildhaus and Dugoni, 2012; Simoni et al., 2012; Acevedo et al., 2015; Hamilton et al., 2015; Mennis and Stahler, 2016), marijuana use is known to be associated with worse health outcomes among African American compared to White Americans (Watts and Wright, 1990; Pacek et al., 2012; Peters et al., 2014).

Marijuana use and depression tend to be comorbid among adolescents (Medina et al., 2007; Repetto et al., 2008; Crane et al., 2015). Individuals who use marijuana (Brown et al., 1996; Degenhardt et al., 2003; Volkow, 2004; Libby et al., 2005) and other substances such as tobacco (Boden et al., 2010; Weinstein and Mermelstein, 2013) report higher levels of depressive symptoms than non-users. Although some research has shown that marijuana use and depressive symptoms may have reciprocal effects on each other (Degenhardt et al., 2003; Patton et al., 2005), most of the existing knowledge is from studies with predominantly White samples (Degenhardt et al., 2003; Patton et al., 2005; Crane et al., 2015). Therefore, there is a need to study these associations specifically among African American adolescents (Kandel et al., 1997; Repetto et al., 2008; Caldwell et al., 2016).

Although recent evidence has suggested that the link between depressive symptoms and marijuana use may differ based on gender (Crane et al., 2015), less is known about such gender differences among African American adolescents (Kandel et al., 1997; Wallace et al., 1999; Repetto et al., 2008). Knowledge about between- and within-group differences is essential for finding racial/ethnic specific and culturally relevant solutions for prevention and control of common mental and behavioral comorbidities associated with marijuana use among male and female African American adolescents (Repetto et al., 2008).

The link between marijuana use and depression can be explained by both psychosocial as well as biological mechanisms. Among various psychosocial theories that explain the link between substance use and depression, Negative Effects Model suggests that depression is a consequence for marijuana use. In this view, behavioral maladjustment (e.g., marijuana use) precedes affective problems (Costello et al., 1999; Armstrong and Costello, 2002). Baseline substance use in this perspective is associated with negative social interactions that increase subsequent risk of depression (Aseltine and Gore, 1998). In a study, Repetto et al. (2008) found that depressive symptoms precede marijuana use among male African American adolescents.

There are also biological explanations for this link. The endogenous cannabinoid system (ECS) is distributed throughout several brain regions including prefrontal cortex, hippocampal regions, as well as white matter (Romero et al., 1997, 2002; Iversen, 2003). Animal studies have documented cellular effects of marijuana use in hippocampal regions (Stiglick and Kalant, 1985; Childers and Breivogel, 1998; Pistis et al., 2004). Human studies have also documented structural brain changes associated with marijuana use (Verdejo-García et al., 2004). Damage to the cannabinoid system may result in depressive-like symptoms (Martin et al., 1996). Some Aasly et al. (1993) and Matochik et al. (2005) but not all Block et al. (2000) and Wilson et al. (2000) human studies have found gray and white matter abnormalities among marijuana users. In a study, marijuana use interacted with white matter volume in predicting depressive symptoms. In marijuana users, but not in controls, white matter volume was inversely associated with depressive symptoms (Medina et al., 2007). In another study, marijuana use interacted with gender on brain measures on the size of prefrontal cortex, with male marijuana users showing a smaller prefrontal cortex volume compared to other groups (Medina et al., 2009).

There is some evidence suggesting that gender may moderate these links. Most research on gender differences in the link between depressive symptoms and marijuana use, however, has been conducted in predominately White populations. African American males and females differ in using behavioral coping to environmental stress (Jackson et al., 2010). While African American males may turn to substance use (Assari et al., in press) and depression (Assari et al., 2017), African American females may have a higher tendency for over eating and obesity (Assari, 2018). As a result, we expect a stronger association between depression and substance use for males than females. In addition, some research has suggested that family support and family SES may have different implications for the health of male and female African American adolescents (Assari et al., 2018). For instance, in one study, maternal support predicted depressive symptoms and obesity for male and female African American adolescents, respectively (Assari et al., 2015).

Building on previous studies on the same topic (Repetto et al., 2008; Crane et al., 2015), the goal of the current study was to explore gender differences in the bidirectional relationships between marijuana use and depression symptoms among African American adolescents. Structural equation modeling (SEM) allows us to test reciprocal associations between marijuana use and depressive symptoms over time. We hypothesized that baseline marijuana use would be related to an increase in symptoms of depression, especially for males (*Hypothesis 1*). We also hypothesized that symptoms of depression at baseline will predict more marijuana use among males (*Hypothesis 2*).

## Materials and Methods

### Study Design and Setting

Using a longitudinal design, this is a secondary data analysis of the first four waves of a parent study, the Flint Adolescent Study (FAS). The FAS is a 12-wave longitudinal study (over a period of 18 years) of adolescents from adolescence through their transition to early adulthood.

### Sampling

All participants were enrolled from Flint, MI, an economically challenged city in the Midwest of the United States. The original FAS enrolled 850 9th graders, in collaboration with the Flint Community Schools. Participants were sampled from four local public high schools. As the primary goal of the study was school dropout, students with a grade point average (GPA) of 3.0 and below were selected. All participants were 9th graders at the start of the study. Having a defined diagnosis of developmental or emotional disability/impairment were exclusion criteria. The study followed the adolescents during their high school years. The study had a 90% response rate from Waves 1 to 4. Mean age of the participants at baseline was 15 years. The FAS sample had an almost equal number of males and females, and was reflective of the overall student body in the Flint High Schools in the Fall in the year 1994 (Aiyer et al., 2014; Assari et al., 2014). Six hundred eighty-one (681) African American adolescents (335 males and 346 females) were included in the current study. Non-African American individuals were excluded from the analytic sample. Age, gender, and socioeconomic status were measured at baseline (9th grade), maternal support and friends’ drug use was collected at Wave 2, and marijuana use and depressive symptoms were measured at Wave 2 (10th grade), Wave 3 (11th grade), and Wave 4 (1 year after high school).

### Ethics

Written informed consent was obtained from parents of participating adolescents. All participants provided written consent or assent before each interview, depending the age of the participant at the time of the interview. The study protocol was approved by the University of Michigan (UM) Institutional Review Board.

### Procedures

Face-to-face interviews were used in the data collection. The interviews were conducted either at school or at an alternative community location such as church. Interviews lasted about 1 h on average. Data collection continued regardless of the school dropout status of the participants.

### Measures

#### Covariates

Age (continuous measure), gender (female vs. male, with male as the reference category), and family socioeconomic status were measured at baseline. Family socioeconomic status was defined as parental employment status and family structure. SES (parental employment) was self-reported. SES was operationalized as a dichotomous variable (both parents employed vs. less than two parents were employed). Family structure was also conceptualized as a dichotomous variable (both biological parents available vs. any other conditions) (Appendix Table A1).

#### Maternal Support

In this study we only used maternal, not paternal, support because a large proportion of adolescents did not report information about their biological fathers due to adolescents not knowing them, not having any real relationship with them, or because they were either in prison or deceased. This is partially due to the fact that only 25% of the participants were living in an intact family at baseline. The maternal support measure in the current study was the modified measure for parental support developed by Procidano and Heller (1983). This measure had five items that assessed closeness of the parent-adolescents relationship as well as the amount that parent provided emotional and instrumental support for the adolescents. Items were on a five-point Likert scale, ranging from 1 (not true) to 5 (very true). A total score was created by calculating an average of all five items (Brenner et al., 2011). (Cronbach’s alpha = 0.879).

#### Friends’ Drug Use

Friends drug use was measured by asking 10 questions. Sample items included “how many of your friends (1) smoke marijuana at least once a month? (2) have used cocaine? and (3) have used heroin or morphine?” Item response included a five category response, including none (1), some (2), many (3), most (4), and all (5). Total score was the average of all items, with higher score indicating more drug use among friends. This measure has been previously used (Elkington et al., 2011) (Cronbach’s alpha = 0.869).

#### Depressive Symptoms

Depressive symptoms were measured using the six items of the Brief Symptom Inventory (BSI; Derogatis and Spencer, 1982, 1993; Derogatis, 1977a). These items assess symptoms of depression such as feeling hopeless about the future and having no interest in things during the past week. Items used a five point Likert scale ranging from 1 (not at all uncomfortable) to 5 (extremely uncomfortable). We calculated a total score which was an averaged of all items. BSI has high reliability and validity in adolescents and adults (Derogatis, 1977b; Derogatis and Spencer, 1982, 1993) Cronbach’s alpha was 0.8249 in our sample at Wave 1.

#### Marijuana Use

Borrowing from the Monitoring the Future Study (Bachman et al., 1997; Repetto et al., 2008), marijuana use was measured using three items that assess the number of times that the participant had used marijuana (grass, weed, pot, hashish) in his or her lifetime, last year, and the last 30 days. Response categories included: 0 times; 1–2 times; 3–5 times; 6–9 times; 10–19 times; 20–39 times; and 40 or more times. Total score was the mean of the three items, ranging from 1 to 7, with higher score indicating more use (Repetto et al., 2008). Cronbach’s alpha was 0.897 at Wave 1.

### Attrition Analysis

Attrition analysis revealed that drop out was not at random. Loss to follow up (drop out) was associated with higher age, male gender, not living in an intact family, and having used more marijuana at baseline. Attrition was not associated with parental employment, maternal support, friends’ drug use, and depressive symptoms.

### Data Analysis

Univariate and bivariate analyses were conducted in SPSS 20.0 (IBM Corporation, Armonk, NY, United States). For bivariate analysis, Pearson’s correlation test and also paired samples t-test were used. We used Analysis of Moment Structure (*AMOS* 18.0) for multivariable analysis (Allison, 2002; Arbuckle, 2009). For multivariable data analysis, we used multi-group latent growth curve modeling (LGCM). LGCM is a subtype of structural equation modeling (SEM) (Kline, 2011). We used LGCM rather than traditional SEM because we wanted to model trajectories rather than change (difference between two observations).

In the first step for LGCM, we fitted unconditional models of depressive symptoms and marijuana use. In the next step, we ran a parallel process model with two growth models for depressive symptoms and marijuana use. Then, we added all covariates. In all multi-group models, the group was defined based on gender. We did not constrain the paths across the groups in unconditional or conditional models with or without covariates (Kock, 2014). Fit statistics included were Chi square, *p*-value, the comparative fit index (CFI) [>0.90], the Chi square to degrees of freedom ratio, and Root Mean Squared Error of Approximation (RMSEA) [<0.06] (Tabachnick and Fidell, 1996; Hu and Bentler, 1999; Lei and Lomax, 2005). Standardized path coefficients were reported. AMOS applies the Full Information Maximum Likelihood (FIML) to handle the missing data. *P* < 0.05 was considered as significant.

## Results

Table 1 summarizes descriptive statistics for demographic data, socioeconomic factors, maternal support, friends’ drug use, marijuana use, and depressive symptoms. Paired samples *t*-test showed that marijuana use increased among males and females over time. Paired samples *t*-test did not show, however, any significant change in depressive symptoms in the pooled sample.

**Table 1:** Descriptive statistics overall and by gender.

	All		Male		Female	
	n	%	n	%	n	%
**Socioeconomic Status (SES)**						
Family structure						
Single parent-headed family	511	75.04	237	70.75	274	79.19
Intact family	170	24.96	98	29.25	72	20.81
Parental employment						
Two parents working	307	45.08	160	47.76	147	42.49
Less than two parents working	342	50.22	162	48.36	180	52.02

	**n**	**Mean (SD)**	**n**	**Mean (SD)**	**n**	**Mean (SD)**

Age	681	14.86 (0.65)	335	14.94 (0.66)	346	14.79 (0.62)
Maternal support	639	4.01 (0.96)	311	4.11 (0.86)	328	3.92 (1.04)
Friends’ drug use	633	1.46 (0.43)	306	1.47 (0.44)	327	1.45 (0.42)
Depressive symptoms 1	647	1.85 (0.87)	315	1.72 (0.82)	332	1.96 (0.91)
Depressive symptoms 2	626	1.80 (0.85)	300	1.66 (0.78)	326	1.93 (0.90)
Depressive symptoms 3	613	1.81 (0.93)	291	1.75 (0.92)	322	1.86 (0.93)
Marijuana use 1	636	6.18 (6.29)	310	6.43 (6.55)	326	5.94 (6.03)
Marijuana use 2	611	6.37 (6.50)	292	6.84 (6.94)	319	5.94 (6.03)
Marijuana use 3	601	3.96 (4.13)	284	4.21 (4.37)	317	3.73 (3.89)


### Bivariate Analysis

As Table 2 indicates, in the pooled sample, females were younger, were less likely to live in an intact family, reported lower maternal support, and higher depressive symptoms at baseline than males. Gender was not correlated with marijuana use at baseline. In the pooled sample, age showed a weak positive correlation and living in an intact family showed a weak negative correlation with frequency of marijuana use at baseline. Friends’ drug use showed a strong positive correlation with marijuana use at baseline. Maternal support showed a weak negative correlation with frequency of marijuana use at baseline.

**Table 2:** Correlation matrix among African American adolescents.

	1	2	3	4	5	6	7	8	9	10	11	12
1 Gender (Female)	1	–0.12**	–0.10*	–0.05	–0.10*	–0.02	0.14**	0.16**	0.06	–0.04	–0.07	–0.06
2 Age		1	–0.10*	–0.09*	–0.01	0.11**	–0.05	–0.06	–0.03	0.12**	0.06	–0.02
3 Intact family			1	0.14**	0.10*	–0.04	–0.09*	0.01	–0.06	–0.15**	–0.15**	–0.06
4 Two parents working				1	0.05	–0.07	–0.05	–0.01	0.01	–0.04	–0.02	–0.03
5 Maternal support					1	–0.20**	–0.18**	–0.14**	–0.13**	–0.12**	–0.16**	–0.13**
6 Friends’ drug use						1	0.13**	0.04	0.08*	0.51**	0.39**	0.37**
7 Depressive symptoms 1							1	0.34**	0.23**	0.07	0.06	0.07
8 Depressive symptoms 2								1	0.36**	0.12**	0.12**	0.13**
9 Depressive symptoms 3									1	0.11**	0.09*	0.13**
10 Marijuana use 1										1	0.69**	0.60**
11 Marijuana use 2											1	0.72**
12 Marijuana use 3												1


*^∗^p < 0.05; ^∗∗^p < 0.01.*

As shown in Table 3, age was positively correlated with baseline marijuana use for males, but not for females. Living in an intact family has a weak negative correlation with baseline marijuana use among both male and female adolescents. For both genders, friends’ drug use showed moderate to strong correlation with baseline marijuana use. Among males, maternal support showed a weak positive correlation with marijuana use at baseline. Among females, maternal support was not correlated with baseline marijuana use.

**Table 3:** Correlation matrix among male and female African American adolescents.

	1	2	3	4	5	6	7	8	9	10	11
1 Age	1	–0.10	–0.10	–0.05	0.11	–0.13*	–0.10	–0.07	0.13*	0.05	–0.05
2 Intact family	–0.12*	1	0.15*	0.12*	–0.06	–0.07	0.04	–0.07	–0.18**	–0.19**	–0.07
3 Two parents working	–0.10	0.13*	1	0.13*	–0.12*	–0.11	0.05	0.02	–0.09	–0.04	–0.06
4 Maternal support	–0.01	0.06	–0.02	1	–0.25**	–0.15**	–0.09	–0.01	–0.16**	–0.19**	–0.12*
5 Friends’ drug use	0.12*	–0.02	0.02	–0.16**	1	0.11	0.08	0.03	0.46**	0.40**	0.32**
6 Depressive symptoms 1	0.03	–0.08	0.01	–0.18**	0.16**	1	0.27**	0.19**	0.07	0.12*	0.06
7 Depressive symptoms 2	0.01	0.01	–0.03	–0.16**	0.01	0.37**	1	0.33**	0.15*	0.19**	0.18**
8 Depressive symptoms 3	0.02	–0.05	0.02	–0.21**	0.13*	0.26**	0.39**	1	0.12*	0.11	0.17**
9 Marijuana use 1	0.10	–0.12*	0.01	–0.11	0.56**	0.08	0.11*	0.11	1	0.68**	0.55**
10 Marijuana use 2	0.06	–0.12*	–0.02	–0.15*	0.38**	0.03	0.09	0.09	0.71**	1	0.74**
11 Marijuana use 3	0.01	–0.06	–0.01	–0.15*	0.42**	0.09	0.10	0.10	0.65**	0.70**	1


*Males upper diagonal (in bold), females lower diagonal.^∗^p < 0.05; ^∗∗^p < 0.01.*

### Multivariable Model

**FIGURE 1:**
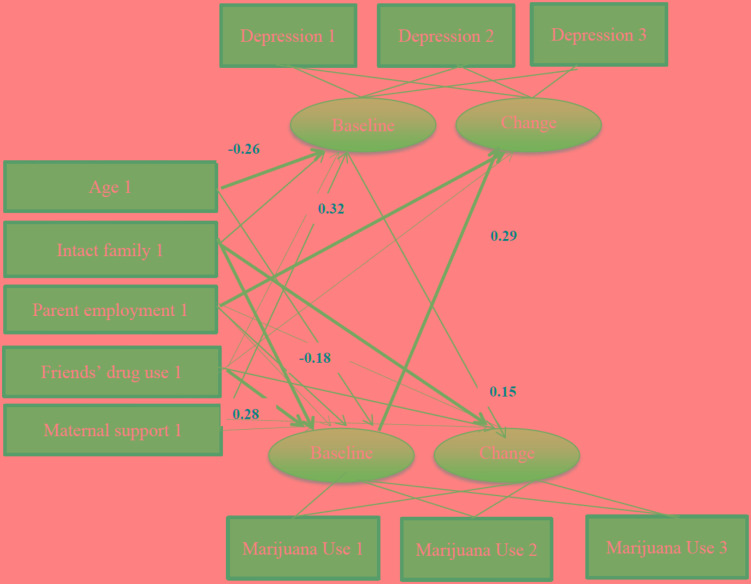
Summary of path coefficients among male African American adolescents [χ^2^ = 138.037, *p* < 0.001, *df* = 40, χ^2^/*df* = 3.451, Root Mean Squared Error of Approximation (RMSEA) = 0.060]. Numbers reflect standardized regression coefficients that are adjusted for other variables. Bold paths reflect significant effects.

**FIGURE 2:**
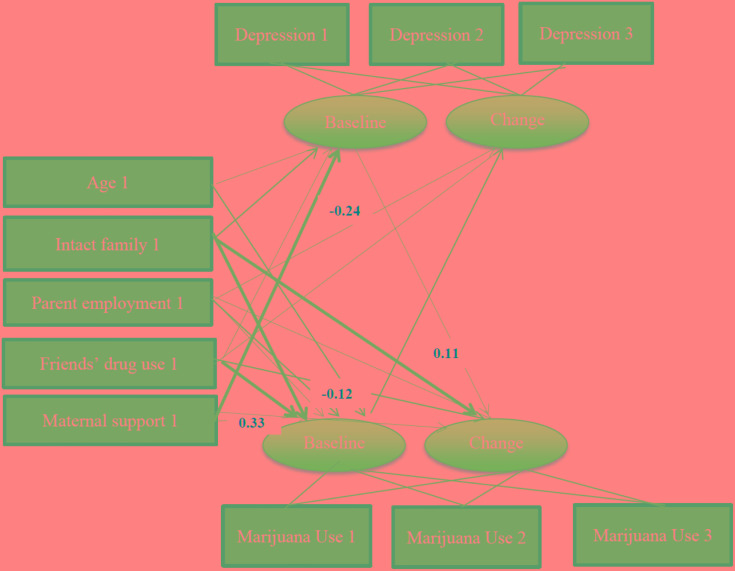
Summary of path coefficients among female African American adolescents [χ^2^ = 138.037, *p* < 0.001, *df* = 40, χ^2^/*df* = 3.451. Root Mean Squared Error of Approximation (RMSEA) = 0.060]. Numbers reflect standardized regression coefficients that are adjusted for other variables. Bold paths reflect significant effects.

The fit of the final multi-group path model was good (χ^2^ = 138.037, *df* = 40, *p* < 0.001, χ^2^/*df* = 3.451, RMSEA = 0.060; Figures 1, 2).

As depicted in Table 4 and Figure 1, among males, there was a significant and positive path from baseline marijuana use to change in depressive symptoms over time. Among males, age was negatively associated with baseline depressive symptoms; having an intact family was associated with lower baseline marijuana use and an increased change in marijuana use over time among male adolescents. Friends’ drug use was positively associated with baseline marijuana use. Maternal support was not associated with baseline or change in marijuana use over time among males.

**Table 4:** Summary of path coefficients among male and female African American adolescents.

			Male		Female	
			Standard (S.E.)	P	Standard (S.E.)	*P*
Age	→	Depressive symptoms (baseline)	–0.26 (0.05)	0.002	0.01 (0.06)	0.875
Intact family (baseline)	→	Depressive symptoms (baseline)	–0.03 (0.09)	0.806	–0.06 (0.12)	0.415
Two parents working (baseline)	→	Depressive symptoms (baseline)	–0.16 (0.09)	0.140	–0.01 (0.10)	0.941
Friends’ drug use (baseline)	→	Depressive symptoms (baseline)	0.08 (0.08)	0.380	0.11 (0.09)	0.079
Maternal support (baseline)	→	Depressive symptoms (baseline)	–0.15 (0.04)	0.078	–0.24 (0.04)	<0.001
Age	→	Marijuana use (baseline)	–0.03 (0.36)	0.504	–0.03 (0.31)	0.450
Intact family (baseline)	→	Marijuana use (baseline)	–0.18 (0.74)	0.001	–0.12 (0.71)	0.023
Two parents working (baseline)	→	Marijuana use (baseline)	–0.04 (0.68)	0.44	0.02 (0.59)	0.731
Friends’ drug use (baseline)	→	Marijuana use (baseline)	0.28 (0.54)	<0.001	0.33 (0.47)	<0.001
Maternal support (baseline)	→	Marijuana use (baseline)	–0.04 (0.28)	0.327	–0.05 (0.20)	0.211
Intact family (baseline)	→	Depressive symptoms (linear slope)	–0.01 (0.07)	0.974	0.05 (0.08)	0.616
Two parents working (baseline)	→	Depressive symptoms (linear slope)	0.32 (0.06)	0.038	0.02 (0.06)	0.857
Marijuana use (baseline)	→	Depressive symptoms (linear slope)	0.29 (0.00)	0.023	0.07 (0.01)	0.406
Intact family (baseline)	→	Marijuana use (linear slope)	0.15 (0.34)	0.003	0.11 (0.32)	0.044
Two parents working (baseline)	→	Marijuana use (linear slope)	0.04 (0.32)	0.432	–0.04 (0.26)	0.510
Depressive symptoms (baseline)	→	Marijuana use (linear slope)	0.06 (0.47)	0.327	–0.02 (0.19)	0.682


As shown in Table 4 and Figure 2, among females, there were no significant paths from baseline depressive symptoms or marijuana use to change in depressive symptoms or marijuana use over time. Age and parent employment were not associated with baseline or change of depressive symptoms or marijuana use. Higher maternal support was associated with lower depressive symptoms at baseline.

## Discussion

Our study had at least three main findings. First, there was a gender difference in the association between marijuana use and depressive symptoms with the association being present only for male, but not female, African American adolescents. Second, baseline marijuana use predicted the trajectory of depressive symptoms over time for males. Third, baseline depressive symptoms were not predictive of change in marijuana use over time for either gender.

Our findings extend the existing literature which is mostly limited to White participants (Rohde et al., 1991; Kandel et al., 1997; Rao et al., 2000; Swendsen and Merinkangas, 2000). Our first result on gender difference in the association between marijuana use and depressive symptoms among adolescents was in line with the results reported by Schuster et al. (2013); Repetto et al. (2008), and Crane et al. (2015) who all documented gender as a moderator for the same association. Our second finding documented paths from baseline marijuana use to subsequent depressive symptoms is in line with the some (Green and Ritter, 2000; Bovasso, 2001; Arseneault et al., 2002; Brook et al., 2002; Fergusson et al., 2002; Patton et al., 2002) but not all (Repetto et al., 2008).

Males and females differ in how they develop comorbid depression and marijuana use (Kandel, 1984; Fergusson and Horwood, 1997; Scaramella et al., 1999; Griffin et al., 2000; Assari et al., 2015; Crane et al., 2015) and African American adolescents are not exception to this rule. In a majority White sample, Crane et al. (2015) found a link between symptoms of depression and marijuana use among 15–16 years old boys, but not girls. In another study by Repetto et al. (2008) of 622 African American adolescents transitioning into young adulthood, baseline depressive symptoms predicted subsequent marijuana in males, while baseline marijuana use did not correlate with future increase in depressive symptoms for females or males.

Our findings suggest that marijuana use has a stronger negative effect on psychological wellbeing of male African American adolescents, compared to their female counterparts. Literature has mentioned that marijuana use may be a coping mechanism for African American males who deal with psychological distress (Schuster et al., 2013) and discrimination (Gerrard et al., 2012). Males are more likely to use external avoidance-based coping strategies, such as substances, to cope with negative affect (Hankin et al., 2007), while females may have a higher tendency to use internalizing coping strategies, such as rumination and isolation (Copeland and Hess, 1995; Hankin et al., 2007). Gender differences in social and environmental influences on marijuana use are also reported (Caggiula et al., 2002; Shrier et al., 2012; Crane et al., 2013). These may be also related to sex differences in brain connectivity (Lei et al., 2016; Conrin et al., 2018).

With some gender differences, parental support was found to protect adolescents against depression and substance use, which is in line with previous research (Wills and Vaughan, 1989; Wills et al., 1996, 2000; Assari et al., 2015). Friends’ drug use also differently influences drug use among boys and girls (Schulte et al., 2009). While conflict with a parent is a risk factor for substance use (Farrell and White, 1998), positive family relations that manifest in different forms such as affection, praise, and encouragement, may lower adolescents’ drug use (Barber, 1992; Knight et al., 1998). The protective effect of parental support against adolescent substance use is shown to be above and beyond peers’ influence (Bahr et al., 1995; Branstetter et al., 2011). Parental support reduces substance use through enhancing self-control and behavioral coping and reducing overall risk-taking tendency and tolerance for deviance (Wills et al., 1996, 2004). The effect of parental support on depression is also mediated by self-evaluation (Valiente et al., 2014).

Although adolescents are trying to individuate themselves from parents during this developmental period, they still need parental support (Settersten and Ray, 2010). The protective effects of parental support on depressive symptoms (Gore and Aseltine, 2003; Miller and Taylor, 2012) and substance use (Ralston et al., 2012) remain during the transition to adulthood. Yet, the links between family resources, depression, and substance use may depend on gender, race, and socioeconomic status (Miller and Taylor, 2012). This study demonstrated the unique role of gender in shaping the effect of parenting on changes in depression and substance use of African American adolescents.

Marijuana use is associated with depression (Degenhardt et al., 2003; Klungsoyr et al., 2006; Mathers et al., 2006; Cuijpers et al., 2007; Hayatbakhsh et al., 2007; van Laar et al., 2007; Pedersen and von Soest, 2009; Brook et al., 2012; CDC, 2012, 2013), particularly in those with early initiation (Silins et al., 2014), however, these links are not constant across genders. Gender influences the prevalence and pattern of marijuana use (SAMSHA, 2013; Johnston et al., 2014) and depression (Hankin and Abramson, 1999; Piccinelli and Wilkinson, 2000; Kuehner, 2003). Understanding how gender moderates the link between symptoms of depression and marijuana use may inform design and evaluation of prevention and treatment programs for African American adolescents.

According to the literature, comorbid depression is associated with earlier onset of drug use (Rohde et al., 1996; Rao et al., 1999), as well as worse drug use outcomes (Rohde et al., 1996; Whitmore et al., 1997) such as delayed recovery (Mallin et al., 2002). Among those who are depressed, comorbid substance use is a predictor of longer depressive episodes (Riggs et al., 1995; King et al., 1996) and higher suicide risk (Bukstein et al., 1993). Extending the literature on how gender alters risk of comorbidity between depression and marijuana use may inform the policies and programs that can be used for prevention and treatment of adolescents that present with comorbid depression and marijuana use.

Our findings are particularly important because marijuana is the most commonly used drug in adolescence (CDC, 2000; Miech et al., 2017) and transition to adulthood (Bachman et al., 1997; Schulenberg et al., 2017) in the United States. These transition periods are the stages of identity development, and are characterized by change and exploration (Arnett, 2002). In these periods, individuals begin managing new roles and responsibilities (Arnett, 2002).

These findings have important implications particularly because of the possibility of an increase in availability of marijuana due to legalization of medical marijuana in the United States (McKenna, 2014; Onders et al., 2015). Marijuana is the most commonly used substance by African American adolescents (CDC, 1998; Miech et al., 2017) and consequences associated with substance use are higher and more devastating for African American adolescents (Wallace and Bachman, 1993). Adolescents may self-medicate using marijuana to cope with the changes and new responsibilities that are a part of their transition into and from adolescence (Chen and Kandel, 1998). Adolescents may also use marijuana under influence of their peers (Dishion and Loeber, 1985; Chabrol et al., 2008). Our findings suggest that African American male adolescents who use marijuana should be screened for depression.

Evidence-based interventions and programs that can prevent substance use in adolescents exist (Griffin and Botvin, 2010). Programs should promote parental support that African American adolescents receive (Caldwell et al., 2004). As substance use in social networks is a main determinant of adolescents’ substance use (Tucker et al., 2015), programs may promote greater involvement of *at risk* adolescents in non-drug using social networks. Evidence-based interventions should be widely implemented in the communities where most at risk adolescents live (Hawkins et al., 2002).

The current study has a few limitations. First and foremost, this study did not enroll a random sample of adolescents. The study did not use a probability sampling framework and the sample was drawn from a single city in Midwest. As a result, our findings are not generalizable to all African American adolescents in the United States. Yet, so few studies have focused on the research questions addressed in the current study. The findings are a useful early step and suggest that more representative sample studies would be a useful next step. Second, the data were old and much has changed regarding psychological impact of marijuana use in adolescents. Legalization of medical marijuana in some US states may have reduced stigma associated with its use. There is a need to replicate these findings in newer data sets. Third, all variables in this study were conceptualized and measured at the individual level, and higher-level ecological factors (availability and acceptability of marijuana, area level socioeconomic status, and racial composition of the neighborhood) were not included. This may be especially important for studies on non-majority adolescents, as experiences of discrimination may weigh on them and influence both their depression and substance use. Nevertheless, our study suggests that individual level factors matter and that examining larger structural factors associated with them may be a vital next step for understanding the process by which individual behaviors manifest themselves. Fourth, our parental support measure was limited to maternal support, as a considerable proportion of our adolescents were not in relation with their fathers. We do know that fathers play a vital role in adolescent development (Salem et al., 1998; Ozer et al., 2013; Caldwell et al., 2014) as well, so future research that specifically includes them would be worthwhile. Yet, the study of mother support adds to our vast knowledge about her vital role in adolescent development. Fifth, attrition was not at random, and individuals who stayed in the study had lower age, were more often females, more frequently lived in an intact family, and used less marijuana at baseline. Despite these limitations, the current study makes a significant contribution to the literature.

## Conclusion

To conclude, the findings presented here revealed that male and female African American adolescents differ in the reciprocal associations between marijuana use and depressive symptoms. Male African American adolescents who use marijuana may be at higher risk of elevated depressive symptoms over time. As a result, male African American adolescents who use marijuana may benefit from combined programs that simultaneously treat depression. Necessity for such combined programs is less evident for female African American adolescents, whose marijuana use and depressive symptoms seem to be unrelated. Maternal support was also differently linked to marijuana use and depressive symptoms among male and female African American adolescents. As a result, male and female African American adolescents may differently benefit from programs that enhance maternal social support.

## Author Contributions

The original idea of this analysis was developed by SA. SA also analyzed the data and drafted the manuscript. MZ designed the main cohort study, acquired the data, and RM, CC, and MZ contributed to all drafts of this manuscript. All authors confirmed the final version of the manuscript.

## Conflict of Interest Statement

The authors declare that the research was conducted in the absence of any commercial or financial relationships that could be construed as a potential conflict of interest.
