# Supplementary material for: Marijuana Use and Depressive Symptoms; Gender Differences in African American Adolescents
Source: Front Psychol. 2018 Nov 16;9:2135. doi: 10.3389/fpsyg.2018.02135 (PMC6250838; doi:10.3389/fpsyg.2018.02135)
Supplement: Supplementary file 1 [file Data_Sheet_1.docx]

Appendix 1: Measurements over three waves in the study

|  | Wave 1 | Wave 2 | Wave 3 |
| --- | --- | --- | --- |
| Age | × | - | - |
| Gender | × | - | - |
| Intact Family | × | - | - |
| Two Parents Working | × | - | - |
| Friends’ Drug Use | × | - | - |
| Parental Support | × | - | - |
| Depressive Symptoms | × | × | × |
| Marijuana Use | × | × | × |
